# The Application of a Performance-Based Self-Administered Intra-procedural Checklist on Surgical Trainees During Laparoscopic Cholecystectomy

**DOI:** 10.1007/s00268-017-4361-4

**Published:** 2017-11-15

**Authors:** Michael El Boghdady, Afshin Alijani

**Affiliations:** Ninewells Hospital and Medical School, University of Dundee, Dundee, DD19SY UK

## Abstract

**Introduction:**

Surgical checklists are in use to reduce errors for safer surgery. We aimed to study the effect of a previously designed performance-based self-administered intra-procedural checklist on the performance of trainees during elective laparoscopic cholecystectomy.

**Methods:**

Twenty-four laparoscopic cholecystectomies were enrolled into the study. Six surgical trainees each performed four procedures, two without the checklist and directly followed by two procedures with the checklist. A soft beeping sound reminded each trainee to apply the checklist every 4 min during the procedures. The unedited videos were analysed using the human reliability analysis technique for the number of consequential errors, number of interventions by the trainer, number of instrument movements and time execution. The trainees’ satisfaction was assessed on a 5-point Likert scale questionnaire. Nonparametric test was used for data analysis. *p* value was defined as significant when *p* < 0.05.

**Results:**

Participants performed statistically better with the application of the checklist compared to when no checklist was used, respectively: Median [IQR] total number of errors 1.51 [0.80] versus 3.84 [1.42] (*p* = 0.002) and consequential errors 0.20 [0.12] versus 0.45 [0.42] (*p* = 0.005), and the number of instrument movements per time decreased from 11.90 [5.34] to 10.38 [5.16] (*p* = 0.04). With the introduction of the checklist, the number of interventions by the trainer per time decreased from 2.79 [1.85] to 0.43 [1.208] (*p* = 0.003). The trainees satisfaction score was 4.5 [1] for the first question, 4 [1] for the second question and 4 [2] for the third question.

**Conclusion:**

The self-administered intra-procedural checklist improved the performance of surgical trainees and decreased the number of interventions by the trainer during laparoscopic cholecystectomy. The trainees were generally satisfied using the checklist during the procedures.

## Introduction

A checklist can be defined as a comprehensive list of important actions or steps to be taken in a specific order. Checklists are used to reduce errors by compensating for potential limits of human memory and attention. It is not believed that checklists prevent all human errors and accidents but checklists can decrease errors if systematically followed [1].

The introduction of a surgical safety checklist by the WHO has significantly reduced the morbidity and mortality in surgery by reducing human errors through pre- and post-procedural evaluations [2]. Other examples of checklists include the advanced trauma life support (ATLS) system [3] and anaesthetic crisis management checklist [4, 5].

There is no previous study in the literature studying the effect of a checklist designed to improve the performance rather than simply acting as aid memoire for the order of the steps of the procedures [6, 7]. In a previously published study, a performance-based self-administered intra-procedural checklist was formulated by consensus among master surgeons who ranked the technical factors influencing the laparoscopic task performance via a link to an online questionnaire. This checklist was then tested in the laboratory environment to improve the task performance of novice surgeons [8]. We aimed to clinically apply the previously developed checklist on the performance of surgical trainees during elective laparoscopic cholecystectomy.

## Methods

Consented senior surgical trainees in general surgery at a major teaching hospital were included in this study to perform consecutive laparoscopic cholecystectomies as primary surgeons. Previous experience was noted from the surgical logbooks. Each surgical trainee had a baseline experience of at least 15 laparoscopic cholecystectomies as the primary surgeon. Each trainee was assisted by a consultant acting as the camera operator during all procedures. Only two trainers were included in this study for standardization.

**Fig. 1:**
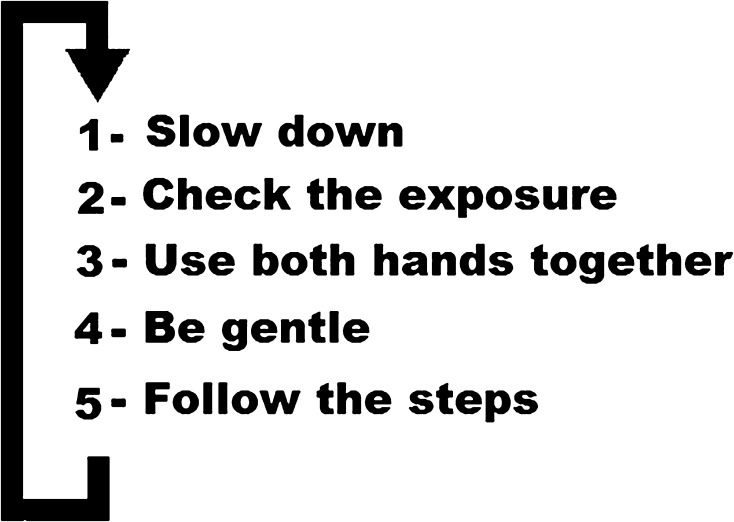
Performance-based checklist

The checklist was piloted prior to the commencement of this study to obtain a power calculation. Each trainee performed two laparoscopic cholecystectomies without the aid of the checklist (Fig. 1) and immediately followed by two further laparoscopic cholecystectomies with the checklist. A negative control study was performed without the application of the checklist. The procedures for each participant were performed within the same working week. To minimize any possible bias resulting from the application of the checklist, the trainees were not told about the existence of the checklist during the control stage, and they were unaware of the aims and nature of this study. Although trainers could not be blinded to the study group with the application of the checklist, they were unaware of the aims of this study including the reasons for the beeping sound and the use of the checklist.

A soft beeping sound was used at 4-min intervals in order to remind the trainees to apply the checklist that was displayed below the laparoscopic monitor (26 in. HD monitor, code 9524NB, colour systems PAL/NTSC, max. screen resolution 1920 × 1200, image format 16:10, power supply 100–240 VAC, 50/60 Hz, Karl Storz).

A standardized setup up was applied for all procedures [9]. The patient was positioned supine with the operating trainee standing on the left side of the patient. A 30 degrees telescope (26003BA, Hopkins^®^, 10 mm diameter, 31 cm length, Karl Storz) was inserted through a 11-mm sub-umbilical trocar. Three further trocars were inserted with 11-mm port in epigastrium, followed by two 5-mm ports, one in the right upper quadrant and one in the right lumbar region of the abdomen. Dissection of Calot’s triangle and the gall bladder were performed using a hook diathermy and a laparoscopic pledget.

All procedures were video-recorded. The unedited video-recordings of the procedures were analysed by a blinded assessor using human reliability analysis technique [10]. A randomly selected number of videos were assessed by second blind assessor as a test of reliability of the results when compared to the first assessor analysis. Human reliability analysis was used for errors, instrument movements and time execution. An error was defined as an action that leads to, or has the potential to lead to, harm to the patient. Error types were identified and classified into consequential and inconsequential errors. Consequential errors were subclassified into (1) perforation of the gall bladder, (2) bleeding-related errors (e.g. bleeding from cystic artery or small vessels) and (3) diathermy burns to surrounding structures (e.g. liver, diaphragm, bowel). Instrument movement was defined as any intra-abdominal unidirectional displacement of the tip of the instrument performed by the primary surgeon.

Each gall bladder was graded (1–3) anatomically as an indication for the potential procedural difficulty [11]. The hierarchal task analysis involved the division of laparoscopic cholecystectomy into three component task zones. Task (1) is the dissection of cystic duct and artery in Calot’s triangle, starting by grabbing the fundus of the gallbladder and ending by insertion of the clip applier. Task (2) is starting by the insertion of the clip applier and ending by the transection of both the cystic artery and cystic duct. Task (3) is the separation of the gallbladder from the liver bed, starting after the transection of both the cystic artery and cystic duct and ending by complete separation of the gallbladder from the liver bed.

The verbal interventions of the trainers directing the trainees during each procedure were recorded and analysed. No attempts were made to match trainees with any specific grade of difficulty for the procedures. Trainees’ satisfaction for using the checklist was subjectively assessed by applying a questionnaire on a 5-point Likert scale at the end of the last procedure performed by each trainee. The survey graded the trainees’ response to the following three statements: (1) I found it easy to apply the checklist, (2) I found the checklist useful, and (3) I will consider using the checklist routinely.

Surgical task endpoints included error numbers, error types, time execution of the procedure, number of instrument movements and number of trainer’s interventions, as well as the trainee satisfaction scores. The surgical task endpoints were standardized for unit time and/or number of instrument movements.

The statistical package for the Social Sciences software (version 22, SPSS Chicago, IL, USA) and Excel (Microsoft^®^ Excel^®^ for Windows 8^®^, Microsoft Corporation, Redmond, WA) were used for data analysis. Data for surgical task endpoints showed nonparametric distribution (median (IQR) Wilcoxon test). *p* value was defined as statistically significant when *p* < 0.05.

## Results

This study was piloted on four laparoscopic cholecystectomies. Total number of errors per total time significantly decreased from 7.16 to 5.37 with the application of the checklist. Based on these results, the power calculation suggested twenty-four cases should enable the detection of 20% difference of median total number of errors with 80% power at 5% level.

Twenty-four laparoscopic cholecystectomies were performed by six surgical trainees each performing four procedures, two before the application of the checklist and directly followed by two procedures with the checklist. All participating trainees had performed at least 15 previous laparoscopic cholecystectomies as primary surgeons. All the participants were right handed, three were male and three female.

When comparing the anatomical grades of difficulty of the procedures in the two groups with and without the application of the checklist, respectively, 5 were graded easy versus 8; 3 graded average versus 3; and 4 graded difficult versus 1.

**Fig. 2:**
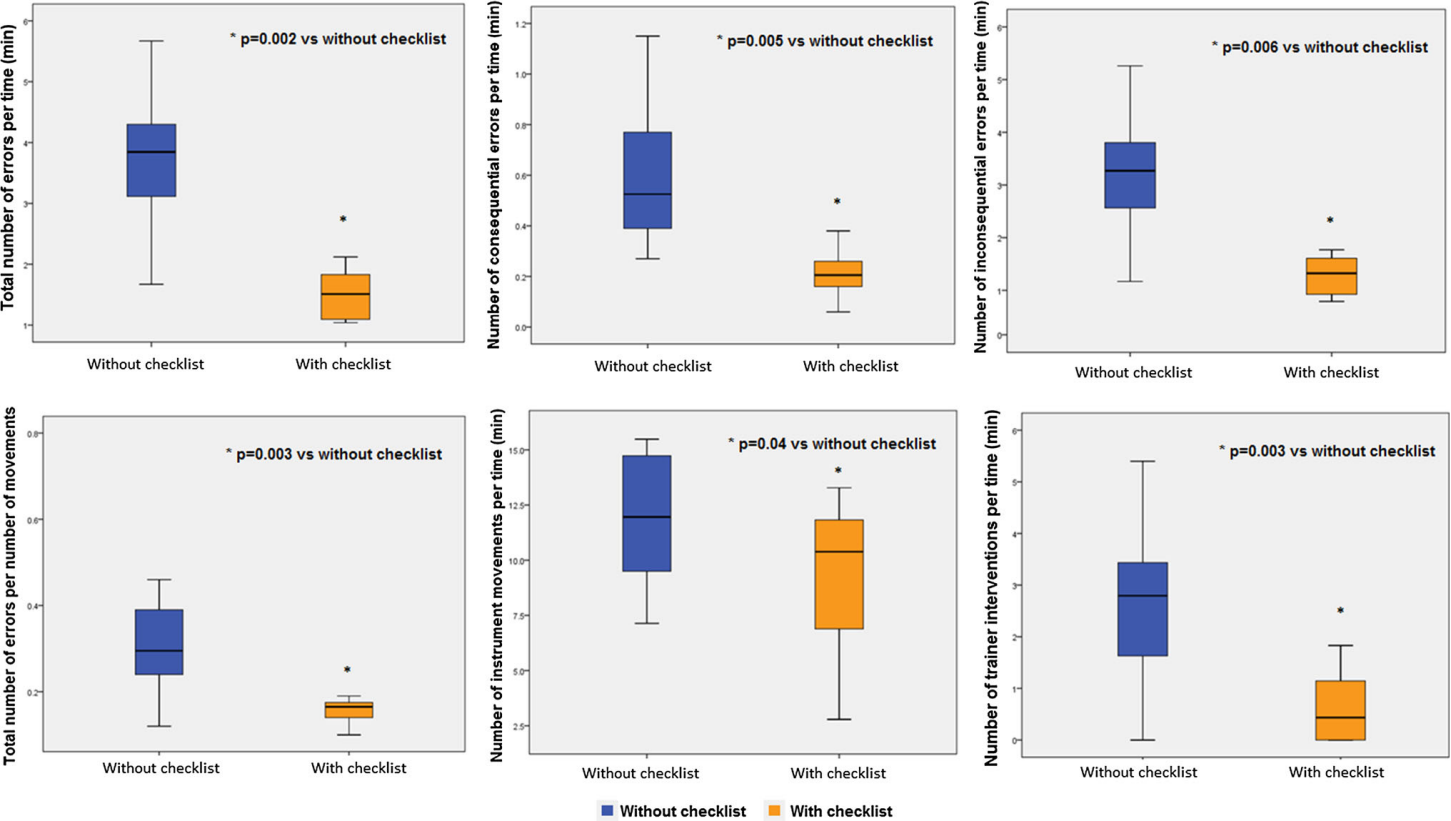
Median (IQR) of the surgical performance without and with the checklist

Participants performed statistically better with fewer number of errors per time with the application of the checklist compared to when no checklist was used, respectively: median [IQR] total number of errors 1.51 [0.80] versus 3.84 [1.42] (*p* = 0.002), consequential errors 0.20 [0.12] versus 0.45 [0.42] (*p* = 0.005), and total number of errors per number of instrument movements 0.1650 [0.04] versus 0.2950 [0.16] (*p* = 0.003), and the number of instrument movements per time decreased from 11.90 [5.34] to 10.38 [5.16] (*p* = 0.04) (Fig. 2). With the introduction of the checklist, the number of interventions by the trainer per time decreased from 2.79 [1.85] to 0.43 [1.208] (*p* = 0.003).

**Fig. 3:**
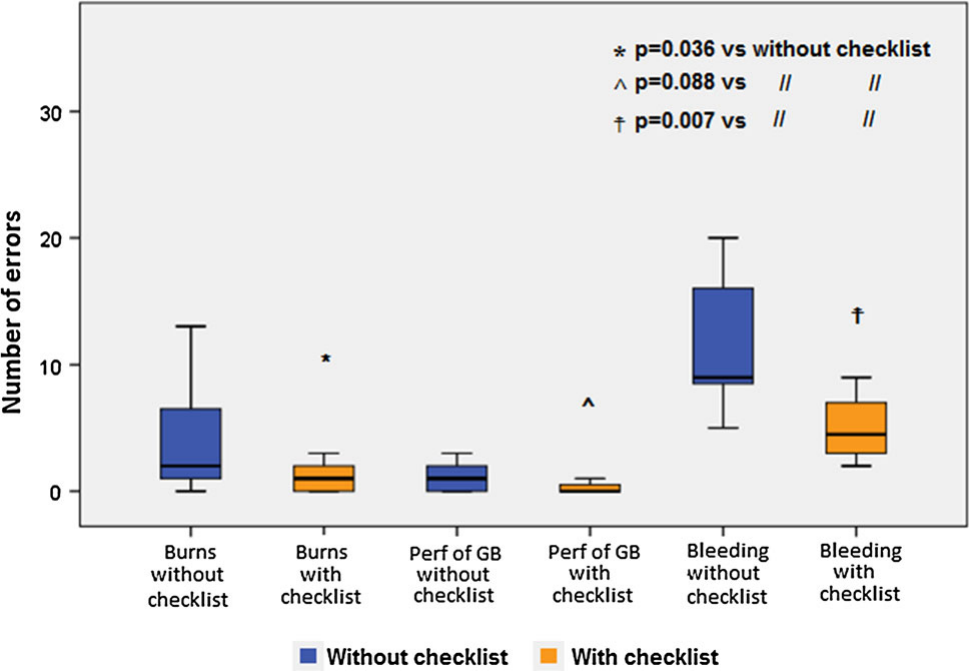
Median (IQR) of the error types without and with the checklist

With the application of the checklist, consequential errors decreased in the perforation of the gall bladder from 1 [2] to 0 [0.75] (*p* = 0.088), bleeding-related errors decreased from 9 [9.75] to 4.5 [5] (*p* = 0.007) and diathermy burns to surrounding structure decreased from 2 [6.75] to 1 [2] (*p* = 0.036) (Fig. 3).

The median [IQR] of the trainees satisfaction score was 4.5 [1] for the first question, 4 [1] for the second question and 4 [2] for the third question.

A negative control study on 8 laparoscopic cholecystectomies from two surgical trainees was performed. Participants showed no statistically significant decrease in the total number of errors in the last 2 procedures when compared to the first procedures, respectively: median [IQR] 1.89 [1.08] versus 2.21 [1.19] (*p* > 0.05).

## Discussion

Our simple performance-based self-administered intra-procedural checklist appears to have a significant accelerating effect on the acquisition of technical skills when clinically applied during elective laparoscopic cholecystectomy. This is the first clinical study to look at a surgical checklist that is simple to be applied, mainly performance based, and used during surgical procedures.

The checklist is short and simple, made of five components making it easy to remember and quick to be applied repeatedly by the trainees. The simplicity of the checklist minimizes its potential interference to distract the participants during the procedure. Previous studies focused on performing the procedure in a step-wise fashion in a correct order. Our checklist included this factor but also included additional important factors that influence the task performance itself. The checklist is based on generic technical factors which makes it applicable to most surgical procedures. Therefore, the application of a mainly performance-based checklist resulted in error reduction rather than error correction by minimizing the occurrence of errors [8].

Non-experienced surgeons tend to operate at the same rate of speed during all the stages of the procedure regardless of its difficulty. It is generally advisable for them to operate at slower rate to reduce the occurrence of errors. Reminding the trainees to slow down through the application of the checklist will cause the desired effect as shown in this study.

An important factor in the performance of surgery is the sufficient exposure. In laparoscopic surgery, exposure is based on intra-abdominal retraction and optical view. Reminding the trainees to check the exposure has the potential advantage of correcting the errors of operating outside the endoscopic view, non-visualization of tip of instrument and weak retraction resulting in poor exposure of the tissues needed for dissection. This may have the effect of decreasing the errors with consequence.

During the intensive concentration required during the performance of laparoscopic tasks, novices and junior trainees often ignore their non-dominant hand at the expense of the dominant one. Reminding the trainees to use both hands optimally has the potential advantage of making the surgeons operate bimanually.

The degree of force applied to the tissue using the instrument is an important independent factor for the performance in laparoscopic surgery, with too little force often resulting in repeating the steps, or too much force causing errors with consequence, such as bleeding or tissue tear. The novices often need guidance throughout the procedure over time in order to understand the appropriate degree of force required to achieve the task. For a non-expert surgeon, it is safer to be gentle in order to minimize any errors with consequence.

Due to the variation in the duration of each procedure, the number of errors, number of instrument movements, error types and number of trainer interventions were calculated per time and/or per instrument movements. Number of errors were calculated per both time and instrument movements. Total number of errors per total number of instrument movements, total number of errors per time, as well as the number of consequential errors significantly decreased after the application of the checklist.

Number of interventions by the trainer per time significantly decreased during the application of the checklist. Since the trainer guidance is regarded as the gold standard, the verbal intervention of the trainer can be seen as a test of external validity for the checklist.

Number of instrument movements per time significantly decreased resulting in improvement in the economy of movement with the application of the checklist. Participants performed the task more accurately and with less number of movements when they tended to slow down, as in general the accuracy of a movement tends to decrease when its speed increases above a threshold. Our interpretation is that slowing down could give the participants more time for visual feedback [12]. The participants’ satisfaction survey indicated the general acceptance of the checklist by the trainees finding it useful and easy to be applied.

Video-assessors were unaware to the existence of the checklist during the analysis of the recordings. This blinding took away any bias of the assessors in analysing the videos for task performance. The application of the checklist did not necessitate any significant pause during the procedures. Furthermore, since any pause due to the application of the checklist was embedded in other natural pauses during lengthy procedures by the trainees, the assessors could not distinguish between the two study groups.

The main limitation of this study was testing the effect of the checklist during laparoscopic cholecystectomy procedures in one centre and in an elective setting. We believe we need to replicate these results in larger studies, in different surgical environments and in other surgical procedures performed by a variety grade of surgeons.

Because of the non-obtrusive and simple format of the checklist, we envisage that trainees will be able to apply it on their own or simply prompted by the trainer. After completing the initial standardized training, the checklist can be applied subconsciously from memory without the need of displaying it for viewing during every procedure. The effect of the checklist on the acquisition of laparoscopic skills during other laparoscopic procedures could be the subject of future studies.

## Conclusion

The self-administered intra-procedural checklist improved the performance of surgical trainees and decreased the number of interventions by the trainer during laparoscopic cholecystectomy. The trainees were generally satisfied using the checklist during the procedures.
